# Cannula-Associated Deep Vein Thrombosis After Extracorporeal Life Support: A Prospective Diagnostic Study

**DOI:** 10.3390/jcm14207241

**Published:** 2025-10-14

**Authors:** Alexander Hermann, Jannis Krais, Anna-Maria Tremetsberger, Robin Ristl, Johannes Philipp Klaeger, Christian Schoergenhofer, Nina Buchtele, Bernhard Nagler, Peter Schellongowski, Oliver Robak, Alexandra-Maria Stommel, Thomas Staudinger

**Affiliations:** 1 Intensive Care Unit 13i2, Department of Medicine I, Medical University of Vienna, Waehringer Guertel 18-20, 1090 Vienna, Austria; jannis.krais@gmail.com (J.K.); anna-maria.tremetsberger@gmx.at (A.-M.T.); nina.buchtele@meduniwien.ac.at (N.B.); bernhard.nagler@meduniwien.ac.at (B.N.); peter.schellongowski@meduniwien.ac.at (P.S.); oliver.robak@meduniwien.ac.at (O.R.); thomas.staudinger@meduniwien.ac.at (T.S.); 2 Center for Medical Data Science, Medical University of Vienna, Spitalgasse 23, BT88, 1090 Vienna, Austria; robin.ristl@meduniwien.ac.at; 3 Department of Pathology, Medical University of Vienna, Waehringer Guertel 18-20, 1090 Vienna, Austria; johannes.klaeger@meduniwien.ac.at; 4 Department of Clinical Pharmacology, Medical University of Vienna, Waehringer Guertel 18-20, 1090 Vienna, Austria; christian.schoergenhofer@meduniwien.ac.at; 5 Department of Emergency Medicine, Medical University of Vienna, Waehringer Guertel 18-20, 1090 Vienna, Austria; alexandra-maria.stommel@meduniwien.ac.at

**Keywords:** critical illness, deep vein thrombosis, extracorporeal membrane oxygenation, pulmonary embolism, venous thromboembolism

## Abstract

**Background:** Venous thromboembolism (VTE), encompassing deep vein thrombosis (DVT) and pulmonary embolism (PE), serves as a major complication in critically ill patients receiving extracorporeal life support (ECLS). The primary aim of the study was to systematically determine the prevalence of cannula-associated DVT following ECLS in a mixed adult ICU population. **Methods:** In this prospective diagnostic study, performed at two ICUs at a tertiary university hospital, we included 101 patients undergoing ECLS between 2016 and 2021. DVT was assessed by vascular ultrasound within 72 h after decannulation or through post-mortem examination. PE was identified by computed tomography when clinically indicated or through post-mortem examination. Both univariate analysis and multivariable logistic regression were used to evaluate risk factors. **Results:** The overall prevalence of DVT was 35%, and PE was found in 9% of patients. PE was significantly more frequent in patients with DVT compared to those without DVT (23% vs. 2%, *p* < 0.001). Logistic regression suggests venovenous configuration as an independent risk factor for DVT compared with venoarterial ECLS (OR = 0.12, 95% CI: 0.04–0.39, *p* = 0.0004). There were no significant differences in coagulation parameters, including anticoagulation target values, in patients with and without DVT. **Conclusions:** This study reveals a considerable prevalence of DVT in patients following ECLS, with VV configuration emerging as a considerable risk factor. PE was common, underscoring the need for routine screening protocols and tailored thromboprophylaxis in this population.

## 1. Introduction

Hospital-acquired venous thromboembolism (VTE), categorized as deep vein thrombosis (DVT) or pulmonary embolism (PE), contributes to morbidity and mortality [1]. Intensive Care Unit (ICU) patients are at particular risk of VTE due to underlying illness, immobility, and the use of intravascular catheters [2]. In Extracorporeal Life Support (ECLS), cannulation of large veins adds endothelial injury, flow disturbance, and delayed mobilization, further increasing thrombotic risk [3,4].

Data on VTE events at the cannula entry site during or after ECLS remain limited, and systematic studies evaluating their impact on mortality are scarce. Most prior investigations on VTEs during ECLS have been retrospective [5], lacked postmortem assessment [6], or focused on specific conditions such as post-cardiotomy patients [7,8], thereby limiting generalizability. Thus, reported VTE rates in patients following ECLS vary widely, ranging from 15% to 85%, reflecting differences not only in study populations but also in clinical standards, screening protocols, diagnostic methods, and anticoagulation strategies [5,8,9,10,11,12,13,14,15,16]. When it comes to the significance of PE in this patient population at risk, our knowledge is particularly limited. Previous estimates suggest PE rates of approximately 23% after ECLS [12,14].

To our knowledge, the present study is the first prospective investigation in a mixed ICU population following ECLS to systematically assess DVT prevalence through ultrasound screening and post-mortem analysis, including approximation of PE prevalence using a combined approach with clinically indicated computed tomography (CT) scan.

## 2. Materials and Methods

### 2.1. General Methods

We analyzed patients in two 8-bed ICUs at the Medical University of Vienna from January 2016 until December 2021 for eligibility. Informed consent was obtained from patients when their power of judgement was present and waived in those who deceased during the ICU stay. Post-mortem examinations were performed if no objection was documented and the next of kin gave verbal or written consent. The study protocol was approved by the ethics review board according to Austrian law regulations (1737/2016).

We included patients treated with venovenous (VV) or venoarterial (VA) extracorporeal membrane oxygenation (ECMO), as well as those with temporary right ventricular assist device (RVAD), provided that at least one cannulation through jugular, subclavian, or femoral vein was present. In our institutional practice, ECLS pump speed was regularly adjusted to 60–80% of estimated cardiac output in VV ECLS, and to ensure adequate preload and organ perfusion in all other configurations. Generally, blood flow is maintained above 2 L/min throughout ECLS, with weaning protocols predominantly involving sweep gas flow adjustments, apart from specific situations with short-lasting blood-flow reductions such as system exchanges or diagnostic assessments.

Inclusion criteria were successful decannulation or death during ongoing ECLS. Exclusion criteria were previously known VTE, pregnancy, and age < 18 years. When the patient was successfully weaned off ECLS, sonographic screening of all prior cannulated veins was performed within the first 72 h after decannulation. As systematic PE screening was clinically and ethically unfeasible, the occurrence of PE by clinically indicated contrast-enhanced CT and structured post-mortem examinations was combined for estimation.

### 2.2. VTE Detection

For the systematic detection of DVT at the antecedent cannulation site, basic b-mode imaging, including color duplex ultrasound and compression, was conducted with a linear probe (5–13 MHz) following the European Society of Intensive Care Medicine (ESICM) recommendations [17]. All scans were performed by a certified intensivist trained in vascular ultrasound. Interpretation was done in consensus with the ICU team. We defined an arbitrary timeframe of 72 h following decannulation for the screening process.

Screening was standardized: veins of the antecedent cannulation were scanned in transverse and longitudinal views, with thrombi identified as hyperechogenic intraluminal material. Absent Doppler flow, abnormal compressibility, or color flow interruption were considered additional evidence of DVT.

For PE detection, a contrast-enhanced CT scan was performed when clinical suspicion was present, assessed by standard of care, including laboratory, echocardiography, clinical signs, and physician judgment. In deceased patients, post-mortem examinations were conducted whenever feasible.

### 2.3. Post-Mortem Examinations

When death occurred during ongoing ECLS, post-mortem examination was intended if no objection had been documented and consent from next of kin was obtained, in line with institutional practice. All but one were performed by the same pathologist. Standard autopsy included macroscopic evaluation of body cavities, organs, and major vessels. To prevent cannula movement and thrombus dislodgement, vessels and cannulas were secured with a thread. Thrombus formation was assessed macroscopically for consistency, composition, and wall adherence to exclude post-mortem coagulation, and confirmed by histology (H&E staining). Pulmonary emboli in central and peripheral arteries were evaluated macroscopically.

### 2.4. Anticoagulation Regime

Our department’s standard of anticoagulation for ECLS patients adheres to the ELSO recommendations [18]. Systemic anticoagulation with unfractionated heparin (UFH) was routinely used, titrated per protocol to a validated anti-Xa assay of 0.2–0.3 IU/mL, measured twice daily or after dose changes [19]. Anticoagulation was paused with bleeding risk (e.g., PLT < 50 G/L) and replaced with pneumatic compression. In cases of heparin-induced thrombocytopenia, Argatroban was used, titrated to diluted thrombin time (hemoclot™ assay) 0.6–0.8 mcg/mL.

Systemic anticoagulation was stopped 1 h before cannula removal. If no bleeding occurred, it was resumed at the previous dose 1 h after removal and maintained for the 24 h compression dressing period according to our institutional standards.

Anti-PLT therapy was added when indicated (e.g., after coronary stenting) without altering anticoagulation. In case of bleeding, both therapies were reassessed and adapted at the physician’s discretion.

### 2.5. Local Management

When percutaneously placed, the cannula was removed, followed by manual compression for 5 min. Surgical removal of the ECLS cannula was performed when the antecedent placement was done surgically by means of an open cutdown with semi-Seldinger technique, or when ongoing local bleeding advised a surgical intervention such as vascular overstitching. For further bleeding prevention, a compression patch (Safe-Guard^®^-Druckpflaster, Maquet, Getinge Group, Rastatt, Germany) was subsequently used for up to 24 h regardless of the respective removal technique.

### 2.6. Documentation

Data documentation was indirectly patient-related and included sonography, CT, and post-mortem reports, as well as demographics, comorbidities, admission reason, ECLS indication, Simplified Acute Physiology Score II (SAPS II) calculated at ICU admission, ICU and hospital survival, length of stay (LOS), ECLS duration, device and cannula details, anticoagulation, routine labs (first daily values), and vital parameters before and after cannula removal.

### 2.7. Statistical Analysis

For the description of demographic data, patient characteristics and outcome variables, the median, 1st quartile and 3rd quartile were calculated for metric variables and absolute and relative frequencies were calculated for categorical variables. An exact two-sided 95% confidence interval (CI) was calculated for the overall prevalence rate of DVT. Wilcoxon rank-sum test and Fisher’s exact test were used for comparisons between patients with and without DVT. Multivariable logistic regression was used to jointly assess the effect of variables that were identified as potential risk factors in the univariable comparisons. For this purpose, only complete cases were used. All calculations were performed using the statistical software environment R version 4.4.

The sample size was planned under the assumption of a DVT prevalence of approximately 25% in patients following ECLS, according to our clinical experience and current evidence [5,6,8,13]. With a sample size of 100, a two-sided 95% confidence interval for a single proportion extends approximately ±8.5 percentage points from the observed proportion for an expected proportion of 25%, which was considered as sufficient statistical precision.

## 3. Results

### 3.1. Population

Of 138 patients assessed for eligibility, 37 were excluded due to contraindications for post-mortem analysis, futile ultrasound, or declined participation, leaving 101 for statistical analysis.

The study population, predominantly male (71%), had a median BMI of 26.1 (IQR 23–30) and a median SAPS II of 42 (IQR 34–52). ECLS configuration was VA in 55%, VV in 41%, and RVAD in 4%, respectively. A single-site venous double lumen cannula was used in 12% of all cases, equaling 28% of all VV configurations (*n* = 12), whereas all others had dual-site cannulation with femoro-jugular flow direction. Further baseline characteristics are depicted in Table 1. The overall median ECLS runtime was 10 days (IQR 5–17). ICU and hospital survival of our cohort was 68% and 67%, respectively. The main indications for ECLS were hypoxic respiratory failure (39%), cardiogenic shock (34%), hypercapnic respiratory failure (7%) and extracorporeal cardiopulmonary resuscitation (ECPR) (7%). Right heart failure, including LVAD-associated RHF, accounted for 6%. Less frequent indications included protected PCI (2%) and septic shock with hypoxic respiratory failure (1%). COVID-19 was the underlying illness in 14% of cases. Previous medical conditions, primary reasons for ICU admission, and indications for ECLS are listed for each patient in Supplemental Table S1.

### 3.2. DVT Prevalence

The overall prevalence of DVT was 35% (*n* = 35, 95% CI 25–45%). Patients with DVT were significantly younger (49 (43–58) vs. 56 (47–64) yrs, *p* = 0.03) and showed a lower severity of illness expressed by SAPS II (40 (30–47) vs. 44 (34–54) *p* = 0.03). There was a significant association of DVT with longer ECLS runtime (8 days vs. 13 days, *p* = 0.02) and VV configuration (*p* < 0.001). DVT occurred more frequently in patients with percutaneous drainage cannula placement (47%) than in those with surgical placement (23%), and percutaneous access was more common among patients who developed DVT (68% vs. 41%; *p* = 0.047), as depicted in Table 1. Among the twelve patients with a double-lumen cannula, six (50%) developed a DVT. Postmortem examinations were performed in 13 patients, with DVT identified in five cases, three of which were also associated with PE.

ICU survival of patients without DVT was not significantly different from survival of patients with DVT (67% vs. 71%, *p* = 0.66). Similar results were found with respect to hospital survival (67% vs. 69%, *p* = 1). Details are outlined in Table 2.

### 3.3. PE Prevalence

The overall prevalence of PE was 9% (*n* = 9). PE was detected significantly more often in patients who also had DVT, indicating a significant association between DVT and PE (8 vs. 1, *p* < 0.001). A total of 37 contrast-enhanced CT scans were performed for PE assessment. In patients with DVT detection by ultrasound (*n* = 30), we performed a CT scan in 20 patients. In post-mortem examinations, PE was identified in 60% of cases with DVT (3 of 5), whereas not a single PE was found in patients without DVT (*n* = 8), demonstrating a significant association (*p* = 0.04). Among the patients with COVID-19 as an underlying illness, DVT prevalence was 64.3% and PE prevalence was 28.6%.

### 3.4. Anticoagulation and Anti-PLT Therapy

Ninety-seven patients received UFH according to our clinical standards.

The mean anti-Xa level was 0.15 (IQR 0.12–0.27) in patients without DVT and 0.22 (IQR 0.13–0.26) in patients with DVT (*p* = 0.8). Similar results were found for aPTT (57.8 s without DVT vs. 57.4 s with DVT, *p* = 0.9).

Three patients were switched to Argatroban: two due to heparin-induced thrombocytopenia (HIT) and one due to the need for excessive UFH doses required to reach anticoagulation targets (“heparin resistance”). Among the HIT patients, one developed both DVT and PE, while the other had neither. The patient requiring high UFH doses developed a DVT but showed no PE on CT scan. Four patients had no systemic anticoagulation for ≥24 h due to ongoing bleeding, two of whom were diagnosed with both DVT and PE.

**Figure 1:**
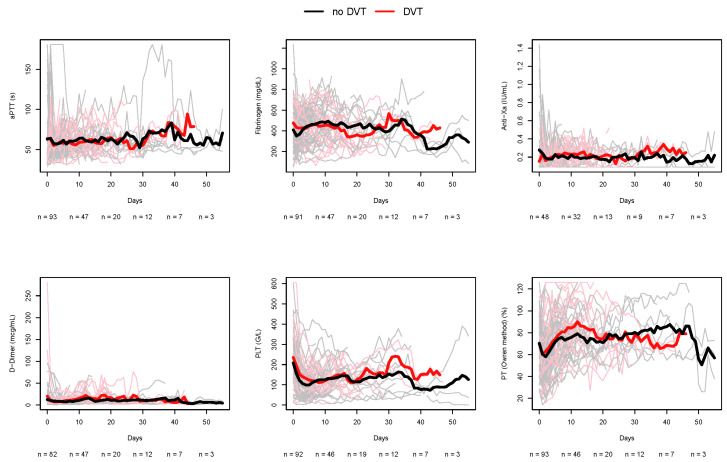
Time trends of selected laboratory parameters during ECLS in patients with and without DVT. DVT—deep vein thrombosis, aPTT—activated partial thromboplastin time, *n*—number, Anti-Xa—Anti-factor-Xa, PLT—platelet count, PT—prothrombin time.

Baseline fibrinogen levels—defined as the closest value measured prior to ECLS initiation—and D-dimer levels throughout the ECMO therapy were significantly higher in patients with later proof of DVT. All other coagulation parameters showed no significant differences, as delineated in Table 3 and Figure 1.

Thirty-one patients received anti-PLT therapy, including acetylsalicylic acid combined with a P2Y12 receptor antagonist in 22 cases and acetylsalicylic acid alone in 9 cases. Among these patients, 16% developed DVT, compared to 84% in the group without anti-PLT therapy, reaching statistical significance (*p* = 0.01). The time-averaged D-Dimer level during ECLS was lower in patients with anti-PLT therapy compared to patients without, with a median of 5.3 (IQR 3.5–8.6) versus 9.6 (IQR 5.9–13.5) mcg/mL (*p* = 0.007).

### 3.5. Multivariate Analysis

The multivariable logistic regression model for DVT, incorporating ECMO runtime, anti-PLT therapy, and configuration, was selected based on their significant associations in univariate analysis and their potential interdependence. It identified a significantly lower risk of DVT for VA ECMO compared to VV ECMO, with an odds ratio (OR) of 0.12 (95% CI: 0.04–0.39, *p* < 0.001). In this model, anti-PLT therapy was non-significantly associated with a reduced frequency of DVT (OR: 0.75, 95% CI: 0.20–2.78, *p* = 0.667). ECMO runtime was not significantly associated with the occurrence of DVT (OR: 0.99, 95% CI: 0.96–1.02, *p* = 0.422), as shown in Table 4.

## 4. Discussion

This study is the first prospective investigation of cannula-related DVT in a mixed ICU population after ECLS, showing a 35% prevalence within 72 h post-decannulation by ultrasound and post-mortem examination.

Three prior studies prospectively analyzed DVT occurrence, two of which, however, exclusively investigated post-cardiotomy populations: among 32 patients treated with VA ECMO without anticoagulation, two cases of DVT were identified [7], whereas autopsy findings in 78 deceased patients revealed venous thrombus formation in 25 cases and systemic thromboembolic events in 24 cases [8]. Another study including a non-ECLS control group found a comparable DVT incidence in critically ill patients without ECLS (33% vs. 32%), suggesting that thrombotic risk may be more closely related to critical illness itself rather than to ECLS exposure alone [6]. Previous investigations in general ECLS populations have otherwise been retrospective, reporting a wide range of DVT frequencies spanning from 10% to 80% [9,10,11,13,20,21,22,23,24,25,26,27].

In univariate analysis, DVT was associated with longer ECLS runtime, VV configuration, and absence of anti-PLT therapy. VA ECLS typically has shorter runtimes and thereby a decreased VTE risk compared to VV mode, reflecting the nature of the underlying conditions [28]. Also, patients on VA ECMO receive anti-PLT therapy more frequently, as coronary interventions often precede their ICU admission. In the adjusted multivariate regression model, fitted to account for potential interplay between these variables, VA ECLS was associated with a reduction in odds of developing DVT compared to VV ECLS, indicating a significant association between VV configuration itself and increased DVT risk. These results are consistent with previous findings suggesting VV ECMO configuration as a categorical risk factor for DVT [29]. However, this finding may also reflect the complex underlying medical conditions of VV ECMO patients, which could independently contribute to increased DVT risk: Apart from the inflammatory response to extracorporeal circulation itself [30], we hypothesize that the higher risk of DVT in VV ECMO patients may, at least in part, reflect hyperinflammatory phenotypes frequently seen in severe ARDS accompanied by a potentially more coagulable state than it might be the case in VA ECLS patients [31,32]. While we did not systematically collect inflammatory biomarkers such as CRP or IL-6, elevated baseline fibrinogen and higher D-dimer levels in DVT patients may indicate enhanced systemic inflammation and immunothrombosis [33], although D-Dimer elevation has been previously reported in patients both with and without VTE [14].

The association of parallel anti-PLT therapy with lower D-dimer levels may indicate a mitigating effect on fibrin turnover. The association of anti-PLT therapy with a reduced prevalence of DVT, however, was not statistically significant within the regression model, suggesting that, in fact, anti-PLT therapy might not have an additive benefit beyond the choice of ECLS configuration. Although the literature suggests that anti-PLT therapy might have the potential to reduce VTE risk in perioperative conditions [34], there is limited evidence supporting the role of add-on anti-PLT therapy in thrombosis prevention during ECLS, aside from specific agents such as prostaglandins [35].

Contrary to previous observations [10,14,29], ECMO duration itself does not appear to significantly influence DVT risk when adjusted for ECLS configuration and anti-PLT therapy in our cohort. This observation underscores the importance of other factors contributing to thrombotic tendency, such as contact activation, factor consumption, mechanical stress, and, as stated above, systemic inflammation [3,4,36].

Baseline fibrinogen levels were higher in patients who later developed DVT, as previously indicated [6]. Other coagulation values showed no differences between the groups, suggesting similar anticoagulation effects. Interestingly, Anti-Xa levels were lower in patients without DVT, though not statistically significant, supporting the trend toward lower anticoagulation targets [37]. Advances in heparin-coated circuits and device design have reduced reliance on systemic anticoagulation [38], though target-controlled management has not been linked to DVT occurrence [10].

The observation that patients with a single-site double-lumen cannula showed a higher DVT prevalence underscores the potential thrombogenicity of this cannulation approach. Literature shows discrepant DVT risks after the use of double-lumen cannulas: While Shafii et al. and Cooper et al. reported a significantly higher DVT occurrence compared to single lumen cannulation [13,24], ELSO registry data, encompassing 11,880 patients (but with barely reproducible DVT detection standards), reports a significantly lower number of thromboembolic events in those using double-lumen cannulas [15]. This raises questions about the mechanical and hemodynamic factors inherent to cannula designs, which may predispose thrombus formation.

PE prevalence was 9% in the entire population and 23% in patients with diagnosed DVT, respectively. These numbers align with previous reports indicating PE occurrence rates of 23.1% [12] and 23.8% [14] following ECLS. Compared to the general ICU population, where autopsy studies report PE in 7–27% of critically ill patients, our findings suggest a comparable or slightly lower PE prevalence in ECLS patients. In our cohort, both DVT and PE were more frequent among patients with COVID-19 as the underlying illness, aligning with previous reports describing PE rates of up to 100% in this population [12].

The significant association between DVT and the presence of PE partly reflects the fact that PE screening by means of contrast-enhanced CT scan was omitted when chances for PE were deemed clinically unlikely. Our findings may therefore be underestimated. However, the association remained significant in post-mortem examinations, despite the small sample size. Notably, no PE cases were observed in patients without DVT during post-mortem examinations, suggesting that DVT may act as a clinical precursor or indicator for PE in this population, as might be expected [39].

Hemodynamic flow conditions are often discussed as a potential contributor to venous thrombosis formation during ECLS, while data on the true hemostatic impact of low-flow situations remain limited. Especially during weaning, practices vary from reducing sweep gas to lowering pump speed or combining both [40]. In our center, flow was generally kept above 2 L/min, with weaning mainly via gas flow reduction to minimize potential risks for stasis-related coagulation. Brief pump reductions or stop-echo assessments were occasionally performed, which, however, further increases variability. Given these confounders, time-weighted flow data was considered misleading and, therefore, not included. Consequently, assessing the true impact of ECLS blood flow on VTE incidence in this heterogeneous population remains inherently challenging and should be addressed in further studies with focused protocols.

Major strengths of our study include the standardized protocol for DVT detection using ultrasound within a defined timeframe post-decannulation, the adherence to an institutional anticoagulation standard, the inclusion of post-mortem examinations to complement in vivo findings, and the application of a comprehensive logistic regression model to adjust for key confounders.

The following limitations should be acknowledged: First, the relatively small sample size and observational design without a control group limit causal relation and generalizability. Second, as only cannula-associated sites were assessed, thrombotic events in other vessels may have been underestimated. Third, additional variables such as SAPS II, baseline fibrinogen, mean D-dimer, inflammatory markers, or age were not included in the multivariable model to avoid overfitting, given the limited number of DVT cases. Whether VV ECMO would remain associated with higher odds of DVT after adjustment for these parameters cannot be determined based on the present data. Finally, whether all DVTs require full anticoagulation regardless of size, wall-adherence, etc., remains unclear, as thrombi were not characterized in detail; future studies should assess risk-adapted strategies.

## 5. Conclusions

Cannula-associated DVT is frequent after ECLS, with VV ECMO carrying a higher risk. These findings underscore the importance of systematic screening and tailored prevention. Future studies should focus on clinical relevance and the full burden of secondary complications such as pulmonary embolism.

## Figures and Tables

**Table 1:** Baseline data.

		*n*	All (*n* = 101)	Overall DVT: No (*n* = 66)	Overall DVT: Yes (*n* = 35)	*p*
Age (yrs)		101	53 (45–62)	56 (47–64)	49 (43–58)	0.031
Sex	male	101	72 (71%)	45 (68%)	27 (77%)	0.368
	female		29 (29%)	21 (32%)	8 (23%)	
BMI		101	26 (23–30)	26 (23–31)	26 (23–29)	0.923
SAPS II		101	42 (34–52)	44 (34–54)	40 (30–47)	0.030
ECLS runtime (d)		101	10 (5–17)	8 (4–13)	13 (7–21)	0.021
Configuration	VV	101	41 (41%)	16 (24%)	25 (71%)	<0.001
	VA		56 (55%)	47 (71%)	9 (26%)	
	RVAD		4 (4%)	3 (5%)	1 (3%)	
Antiplatelet therapy at screening	Y	95	31 (33%)	26 (41%)	5 (16%)	0.012
	N		64 (67%)	37 (59%)	27 (84%)	
Heparin at screening	Y	97	71 (73%)	45 (71%)	26 (76%)	0.639
	N		26 (27%)	18 (29%)	8 (24%)	
Double lumen cannula	Y	101	12 (12%)	6 (9%)	6 (17%)	0.332
	N		89 (88%)	60 (91%)	29 (83%)	
DVT at drainage cannula	Y	89	15 (17%)	0 (0%)	16 (55%)	n.a.
	N		74 (83%)	60 (100%)	13 (45%)	
Drainage cannula placement	Percutaneous	71	36 (51%)	19 (41%)	17 (68%)	0.047
	Surgical		35 (49%)	27 (59%)	8 (32%)	
DVT at reperfusion cannula	Y	89	23 (26%)	0 (0%)	19 (66%)	n.a.
	N		66 (74%)	60 (100%)	10 (34%)	
Reperfusion cannula placement	Percutaneous	71	36 (51%)	19 (41%)	17 (68%)	0.047
	Surgical		35 (49%)	27 (59%)	8 (32%)	
DVT at dual lumen cannula	Y	12	6 (50%)	0 (0%)	6 (100%)	n.a.
	N		6 (50%)	6 (100%)	0 (0%)	
Dual lumen cannula placement	Percutaneous	11	8 (73%)	4 (67%)	4 (80%)	1
	Surgical		3 (27%)	2 (33%)	1 (20%)	

Numbers are given as *n* (%) or n (range) or median (IQR) unless otherwise stated. *n*—number of valid values, *p*—significance level, DVT—deep vein thrombosis, yrs—years, BMI—body mass index, SAPS II—Simplified Acute Physiology Score II, ECLS—extracorporeal life support, VV—venovenous, VA—venoarterial, RVAD—right ventricular assist device, Y—Yes, N—No, IQR—interquartile range, n.a.—not applicable.

**Table 2:** Secondary outcomes.

		*n*	All (*n* = 101)	Overall DVT: No (*n* = 66)	Overall DVT: Yes (*n* = 35)	*p*
ICU survival	Y		69 (68%)	44 (67%)	25 (71%)	
	N	101	32 (32%)	22 (33%)	10 (29%)	0.660
Hospital survival	Y		68 (67%)	44 (67%)	24 (69%)	
	N	101	33 (33%)	22 (33%)	11 (31%)	1
Overall PE	Y		9 (9%)	1 (2%)	8 (23%)	
	N	101	92 (91%)	65 (98%)	27 (77%)	<0.001
Postmortem examination	Y		13 (13%)	8 (12%)	5 (14%)	
	N	101	88 (87%)	58 (88%)	30 (86%)	0.763
PE in postmortem examination	Y		3 (23%)	0 (0%)	3 (60%)	
	N	13	10 (77%)	8 (100%)	2 (40%)	0.035

Numbers are given as *n* (%). *n*—number of valid values, DVT—deep vein thrombosis, ICU—intensive care unit, Y—Yes, N—No, PE—pulmonary embolism.

**Table 3:** Coagulation laboratory parameters during ECLS; comparisons between groups were performed using the Wilcoxon rank-sum test. Baseline refers to the closest value measured prior to ECLS initiation.

		*n*	All (*n* = 101)	Overall DVT: No (*n* = 66)	Overall DVT: Yes (*n* = 35)	*p*
aPTT (s)	Mean	98	57.7 (50.8–64.7)	57.8 (51.1–64.7)	57.4 (51.1–64.4)	0.908
	Baseline	93	47.4 (38.1–61.4)	47.9 (40.0–64.3)	45.2 (37.3–58.1)	0.522
Fibrinogen (mg/dL)	Mean	98	399 (316–522)	399 (314–498)	399 (331–533)	0.509
	Baseline	91	422 (287–564)	359 (252–552)	475 (398–615)	0.035
Anti-factor Xa (UFH-	Mean	69	0.19 (0.12–0.27)	0.15 (0.12–0.27)	0.22 (0.13–0.26)	0.802
assay) (IU/mL)	Baseline	49	0.09 (0.09–0.23)	0.09 (0.09–0.26)	0.1 (0.09–0.21)	0.754
D-Dimer (mcg/mL)	Mean	98	8.1 (4.7–12.9)	7.2 (4.4–10.9)	10.3 (6.0–13.8)	0.049
	Baseline	82	4.6 (2.5–9.7)	4.6 (2.4–10.2)	5.1 (2.9–8.8)	0.930
PLT (G/L)	Mean	98	139 (95–175)	135 (90–167)	146 (103–194)	0.250
	Baseline	92	201 (142–265)	191 (138–255)	219 (157–324)	0.263
TPZ (Owren) (%)	Mean	98	69 (57–82)	67 (55–81)	71 (58–83)	0.397
	Baseline	93	69 (57–86)	70 (50–87)	67 (59–83)	0.832

Numbers are given as median (IQR) unless otherwise stated. ECLS—extracorporeal life support, *n*—number of valid values, DVT—deep vein thrombosis, aPTT—activated partial thromboplastin time, UFH—unfractioned heparin, PLT—platelets, TPZ—Thromboplastin time, IQR—interquartile range.

**Table 4:** Multivariable logistic regression model for DVT with three predictor variables. The table shows the odds ratio for DVT (95% confidence interval) and the *p*-value for a Wald test of the null hypothesis OR = 1.

Variable	Odds Ratio (95% CI)	*p*
Anti-PLT therapy	0.75 (0.20–2.78)	0.667
ECMO runtime	0.99 (0.96–1.02)	0.422
VA vs. VV configuration	0.12 (0.04–0.39)	<0.001
RVAD vs. VV configuration	0.24 (0.02–2.62)	0.244

DVT—deep vein thrombosis, CI—confidence interval, PLT—platelet, ECMO—extracorporeal membrane oxygenation, VA—venoarterial, VV—venovenous, RVAD—right ventricular assist device.

## Data Availability

The raw data supporting the conclusions of this article will be made available by the authors on request.

## Supplementary Materials

The following supporting information can be downloaded at: https://www.mdpi.com/article/10.3390/jcm14207241/s1, Supplemental Table S1: Previous medical conditions, primary reasons for ICU admission, indications for ECLS, and the type of ECLS.

## Abbreviations

The following abbreviations are used in this manuscript:

AF	atrial fibrillation
AMI	acute myocardial infarction
Anti-Xa	anti-factor Xa activity
aPTT	activated partial thromboplastin time
ARDS	acute respiratory distress syndrome
BMI	body mass index
CI	confidence interval
CKD	chronic kidney disease
CPR	cardiopulmonary resuscitation
CS	cardiogenic shock
CT	computed tomography
DVT	deep vein thrombosis
ECMO	extracorporeal membrane oxygenation
ECLS	extracorporeal life support
ECPR	extracorporeal cardiopulmonary resuscitation
ICU	intensive care unit
IQR	interquartile range
LOS	length of stay
n.a.	not applicable
PE	pulmonary embolism
PLT	platelets
PT	prothrombin time
RVAD	right ventricular assist device
SAPS II	simplified acute physiology score II
SD	standard deviation
UFH	unfractionated heparin
VA	venoarterial
VV	venovenous
VTE	venous thromboembolism
Y/N	Yes/No
