# Supplementary material for: Cannula-Associated Deep Vein Thrombosis After Extracorporeal Life Support: A Prospective Diagnostic Study
Source: J Clin Med. 2025 Oct 14;14(20):7241. doi: 10.3390/jcm14207241 (PMC12565285; doi:10.3390/jcm14207241)
Supplement: Supplementary file 1 [file jcm-14-07241-s001.zip › jcm-3898828-supplementary.pdf]

Supplemental Table S1

Previous medical conditions, primary reasons for ICU admission, indications for ECLS, and the type of ECLS.

Patient	Previous medical conditions	Reasons for ICU admission	Indication for ECLS	Type of ECLS
1	COPD, OMF	AECOPD	hypercapnic respiratory failure	VV
2	Histiocytosis X, LUTX 2013	pneumonia	hypercapnic respiratory failure	VV
3	Myocarditis	CS	CS	VA
4	Aortic dissection following RCA coronary angriography	postoperative care (CABG + ascendens and aortic valve replacement)	CS	VA
5	St.p. LTX	RSV pneumonia	hypercapnic respiratory failure	VV
6	Influenza	Influenza pneumonia	hypoxic respiratory failure	VV
7	St.p. LTX	PJP pneumonia	hypercapnic respiratory failure	VV
8	CAD, AMI	st.p. CPR	CS	VV
9	Aspiration, peritonitis, incarcerated hernia	ARDS	RHF and hypoxic respiratory failure	VA
10	PM due to SSS	postoperative care (pericardiocentesis due to cardiac perforation with tamponade)	CS	VA
11	near-drowning	st.p. CPR	hypoxic respiratory failure	VV
12	St.p. LTX	ARDS	hypoxic respiratory failure	VV
13	ICMP, AMI	CS	RHF on LVAD	RVAD
14	Capillary leakage syndrome	shock	CS and hypoxic respiratory failure	VA
15	none	ARDS	hypoxic respiratory failure	VV
16	St.p. LTX	pneumonia	hypercapnic respiratory failure	VV
17	esophageal carcinoma, peritoneal carcinosis, AF	pneumonia	hypoxic respiratory failure	VV
18	EAA, DM II	ARDS	hypoxic respiratory failure	VV
19	epilepsia, iliac artery aneurysm and dissection, polyneuropathy	CS	CS	VA
20	CAD, cAVK, artH	CS	CS	VA
21	aortic dissection type B	pneumonia	hypoxic respiratory failure	VV
22	none	CS	CS	VA
23	toxic liver disease, artH, sleep apnea	Influenza pneumonia	hypoxic respiratory failure	VV
24	Influenza	ARDS	hypoxic respiratory failure	VV
25	artH	st.p. CPR	CS	VA
26	none	postoperative care (mitral valve replacement)	CS	VA
27	artH, AF, DM II, hypothyreoidism	Influenza pneumonia	hypoxic respiratory failure	VV
28	none	Influenza pneumonia	hypoxic respiratory failure	VV
29	granulomatosis with polyangitis and renal involvment	ARDS	hypoxic respiratory failure	VV
30	CAD, ICMP	CS	RHF on LVAD	RVAD
31	CAD, artH, M. Hodgkin	St.p. CPR, pneumonia	hypoxic respiratory failure	VA
32	artH, HLP, MCI	CS	CS	VA
33	CAD, artH, ICMP, MCI	st.p. CPR	CS	VA
34	CLL, aortic valve endocarditis	mixed septic CS	CS	VA
35	St.p. aortic valve replacement	St.p. CPR	CS	VA
36	Ewing's sarcoma	tumor lysis, shock, pneumonia	CS and hypoxic respiratory failure	VA
37	near-drowning	st.p. CPR	hypoxic respiratory failure	VV
38	CAD, artH	postoperative care (aortic replacement)	CS	VA
39	CAD, st.p. CABG-OP, DM II	CS	CS	VA
40	AMI	CS	CS	VA

41	none	st.p. CPR	CS	VA
42	ICMP, artH, DM II, HLP	postoperative care (CABG)	CS	VA
43	hypothermia	st.p.CPR	CS	VA
44	Mycosis fungoides, MR	CS	RHF on LVAD	RVAD
45	St.p. 2x LTX	allograft dysfunction	hypercapnic respiratory failure	VV
46	CAD, AMI	st.p. CPR	ECPR	VA
47	AMI	CS	CS	VA
48	St.p. aortic and mitral valve replacement, prosthetic endocarditis	postoperative care (Re-aortic and mitral valve replacement)	CS	VA
49	lymphoma	diffuse alveolar hemorrhage	hypoxic respiratory failure	VV
50	SLE	MOF	septic shock and hypoxic respiratory failure	VA
51	none	st.p. CPR	ECPR	VA
52	CAD, EAA	ARF	hypoxic respiratory failure	VV
53	SLE, CKD	postoperative care (mitral and aortic valve replacement)	CS	VA
54	none	Influenza pneumonia	hypoxic respiratory failure	VV
55	lung emphysema	ARDS	hypoxic respiratory failure	VV
56	Alcoholic liver disease	CS	CS	VA
57	CAD, COPD, HLP, prostata hyperplasia	CS	CS	VA
58	COPD	AECOPD	hypercapnic respiratory failure	VV
59	aortic valve stenosis, AMI	CS	CS	VA
60	HLP	CS	CS	VA
61	Alcoholic liver disease	septic shock	hypoxic respiratory failure	VV
62	DM II, diabetic PNP	postoperative care (complex PCI)	protected PCI	VA
63	none	septic shock	hypoxic respiratory failure	VV
64	St.p. aortic valve replacement, DCMP	mixed septic CS	CS	VA
65	AMI	st.p. CPR	ECPR	VA
66	PAH	pneumonia	RHF	VA
67	ArtH, DM II, psoriasis arthritis	CS	CS	VA
68	pulmonary emphysema	pneumonia	hypoxic respiratory failure	VV
69	aortic dissection type A, st.p. aortic valve and ascendens replacement	postoperative care (aortic replacement)	CS	VA
70	ICMP, AF, AMI	PJP pneumonia	hypoxic respiratory and circulatory failure	VA
71	COPD, CHF	ARDS	hypoxic respiratory and circulatory failure	VA
72	AMI, CAD	postoperative care (complex PCI)	protected PCI	VA
73	Nephrolithiasis, AMI	CS	CS	VA
74	CAD, artH, DM II, prostate carcinoma, AMI	CS	CS	VA
75	CAD, artH, HLP, DM II	st.p, CPR	ECPR	VA
76	HLP	st.p. CPR	ECPR	VA
77	ICMP, artH	Influenza pneumonia	RHF on LVAD	RVAD
78	COPD, CAD, AMI, infarct-associated VSD	postoperative care (VSD occlusion)	CS	VA
79	none	SCARDS	hypoxic respiratory failure	VA
80	AMI	CS	CS	VA
81	none	Influenza pneumonia	hypoxic respiratory failure	VV
82	VSD	st.p. CPR	ECPR	VA
83	dermatomyositis	pneumonitis	hypoxic respiratory failure	VV
84	systemic mastocytosis	anaphylactic shock	shock and hypoxic respiratory failure	VA
85	none	SCARDS	hypoxic respiratory failure	VV

86	artH, AF, AVB III°	st.p. CPR	ECPR	VA
87	none	SCARDS, myocarditis	hypoxic respiratory and circulatory failure	VA
88	Mb. Parkinson	CS	CS	VA
89	COPD, asthma bronchiale, artH	st.p. CPR	hypoxic respiratory and circulatory failure	VA
90	capillary hemangiomatosis, PAH	st.p. CPR	RHF	VA
91	none	SCARDS	hypoxic respiratory failure	VV
92	none	SCARDS	hypoxic respiratory failure	VV
93	none	SCARDS	hypoxic respiratory failure	VV
94	NSIP	SCARDS	hypoxic respiratory failure	VV
95	steatosis hepatis, thalassaemia minor	SCARDS	hypoxic respiratory failure	VV
96	fibromyalgia	SCARDS	hypoxic respiratory failure	VV
97	none	SCARDS	hypoxic respiratory failure	VV
98	none	SCARDS	hypoxic respiratory failure	VV
99	artH	SCARDS	hypoxic respiratory failure	VV
100	glucose-6-phosphate-dehydrogenase deficiency	SCARDS	hypoxic respiratory failure	VV
101	none	SCARDS	CS and hypoxic respiratory failure	VA

AECOPD - acute exacerbation of chronic obstructive pulmonary disease, AF - atrial fibrillation, AMI - acute myocardial infarction, artH - arterial hypertension, ARDS - acute respiratory distress syndrome, AVB - atrioventricular block, CABG - coronary artery bypass graft, CAD - coronary artery disease, CKD - chronic kidney disease, CLL - chronic lymphocytic leukemia, COPD - chronic obstructive pulmonary disease, CPR - cardiopulmonary resuscitation, CS - cardiogenic shock, cAVK - cerebral arterial vascular disease, DM II - diabetes mellitus type II, EAA - exogenous allergic alveolitis, ECPR - extracorporeal cardiopulmonary resuscitation, ECLS - extracorporeal life support, HLP - hyperlipidemia, ICMP - ischemic cardiomyopathy, ICU - intensive care unit, LTX - lung transplantation, MR - mitral regurgitation, MCI - myocardial infarction, MOF - multi-organ failure, NSIP - nonspecific interstitial pneumonia, OMF - osteomyelofibrosis, PAH - pulmonary arterial hypertension, PJP - Pneumocystis jirovecii pneumonia, PM - pacemaker, PNP - polyneuropathy, RSV - respiratory syncytial virus, RVAD - right ventricular assist device, SCARDS - severe COVID-19-associated ARDS, SSS - sick sinus syndrome, st.p. - status post, VSD - ventricular septal defect, VA - venoarterial, VV - venovenous
